# Karyotype and DNA barcode of *Polyommatus* (*Agrodiaetus*) *cyaneus* (Staudinger, 1899) from its type locality: implication for taxonomic and evolutionary research in *Polyommatus* blue butterflies (Lepidoptera, Lycaenidae)

**DOI:** 10.3897/CompCytogen.v14i4.59574

**Published:** 2020-11-17

**Authors:** Vladimir A. Lukhtanov, Alexander V. Dantchenko, Karine V. Balayan, Anastasia V. Gagarina

**Affiliations:** 1 Department of Karyosystematics, Zoological Institute of the Russian Academy of Sciences, Universitetskaya nab. 1, St. Petersburg 199034, Russia Zoological Institute, Russian Academy of Sciences St. Petersburg Russia; 2 Faculty of Chemistry, Lomonosov Moscow State University, GSP-1, Leninskiye Gory 1/11, Moscow119991, Russia Lomonosov Moscow State University Moscow Russia; 3 Yerevan Botanical Garden of the Institute of Botany of the NAS RA, Acharyan str. 1, Yerevan 0040, Armenia Institute of Botany, NAS RA Yerevan Armenia

**Keywords:** *
Agrodiaetus
*, chromosome, karyosystematics, taxonomy

## Abstract

Chromosomal and molecular analyses of rapidly evolving organisms such as *Polyommatus* Latreille, 1804 blue butterflies are essential for understanding their taxonomy and evolutionary history, and the studies of populations from their type localities are crucially important for resolving problems of nomenclature and species identity. Here we present data on the topotypical population of the blue butterfly species described as Lycaena damone var. cyanea Staudinger, 1899. This taxon was described from Khankendi (Nagorno-Karabakh, Caucasus), and rediscovered at the type locality for the first time since it was collected there in 1869. The specimens were found on dry stony meadows with a predominance of *Onobrychis radiata* Bieberstein, 1810, on upper border of oak forests. Their haploid chromosome number (n) was established as n = 17. Chromosomal and mitochondrial DNA barcode analyses of the studied samples from type-locality provided an opportunity for the critical taxonomic re-examination of Caucasian species of the subgenus Agrodiaetus Hübner, 1822 of the genus *Polyommatus* Latreille, 1804. The obtained data support the interpretation of the P. (A.) cyaneus (Staudinger, 1899) and P. (A.) carmon (Herrich-Schäffer, 1851) as two different, not closely related species complexes as previously hypothesized by Hugo de Lesse. On the contrary, the treatment by Walter Forster who considered these taxa as two groups of conspecific populations was not supported by our data.

## Introduction

The species-rich butterfly subgenus Agrodiaetus Hübner, 1822 of the genus *Polyommatus* Latreille, 1804 has become a model system for studying speciation and chromosomal evolution (Lukhtanov et al. 2005; Wiemers et al. 2009; Dincă et al. 2013; Lukhtanov et al. 2020a). However, despite the attention from biologists, numerous taxonomic and nomenclatural problems remain unresolved in the subgenus. In particular, this concerns the taxon known as P. (A.) cyaneus (Staudinger, 1899), which is a polytypic species (or even a complex of closely related species) (Eckweiler and Bozano 2016). This taxon was initially described as a “variation” Lycaena damone var. cyanea Staudinger, 1899, based on specimens collected in 1866 by Josef Haberhauer in Hankynda (now Khankendi, Nagorno-Karabakh) and in Akhalzich (now Akhaltsikhe, Georgia) (Lederer 1870; Staudinger 1899). In the first detailed revision of the subgenus Agrodiaetus Hübner, 1822, Walter Forster (1956) treated it as a separate genus, designated a specimen from Hankynda as the lectotype of Lycaena damone var. cyanea, and regarded this taxon as subspecies *Agrodiaetus carmon cyanea* (Forster, 1956). However, after the chromosomal studies of Hugo de Lesse (1960, 1963), *Agrodiaetus carmon* (Herrich-Schäffer, 1851) and *A. cyaneus* are usually considered as two different species. At the same time, it is important to emphasize that these studies (de Lesse 1960, 1963) and consequent studies on karyosystematics and molecular taxonomy of the P. (A.) carmon and P. (A.) cyaneus species groups (Wiemers 2003; Lukhtanov et al. 2014) dealt with butterflies from Iran and Turkey and never affected the population from Nagorno-Karabakh.

**Figure 1.**
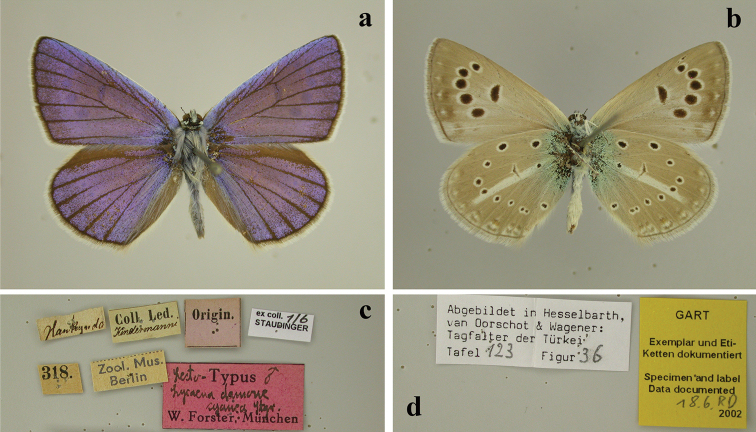
Lectotype of Lycaena damone var. cyanea Staudinger, 1899. In collection of Humboldt-Universität zu Berlin. Photo: V. Lukhtanov **a** upperside **b** underside **c** labels **d** additional labels.

Accordingly to the lectotype designation (Forster 1956), Khankendi in Nagorno-Karabakh is treated as the type locality of *P. cyaneus*. It is generally accepted that the knowledge of karyotype characters of topotypical populations is an essential requirement for revealing species identity in the subgenus Agrodiaetus (Lukhtanov and Dantchenko 2002a; Kandul et al. 2004). As it was shown, the cytological approach using DNA data for certain type populations led to dramatic taxonomic rearrangements on the species level (Lukhtanov et al. 2006, 2008, 2015; Lukhtanov and Dantchenko 2017). In the case of *P. cyaneus* such study of the population from Nagorno-Karabakh seemed especially important because the study of the lectotype specimen (Fig. 1) revealed that the latter differed significantly from the Iranian and Turkish butterflies (e.g. see the figures in Hesselbarth et al. 1995; Eckweiler and Bozano 2016), which were previously attributed to this species.

Here we present the first karyotype description of P. (A.) cyaneus exactly from its type locality. As suggested previously (Lukhtanov and Iashenkova 2019), we also provide the DNA barcodes for the chromosomally studied samples to avoid the possible problems of inaccurate species identification.

## Material and methods

The specimens of P. (A.) cyaneus (5 males and 2 females) were collected by the third author, Karine Balayan, in vicinity of Stepanakert (Khankendi, Nagorno-Karabakh) and near Kanachtala village (20 km to the west from Stepanakert). The collection of the specimens was carried out during July of three summer seasons: in 2015, 2016 and 2018. The collecting places are dry stony glades in oak forest with dominating *Onobrychis radiata* Bieberstein, 1810 (Fabacaea). For chromosomal analysis, testes were extracted from the butterfly abdomens and fixed in a mixture of glacial acetic acid and 96% ethyl alcohol (1: 3). The fixed material was stored at + 4 °C for 5–24 months. For molecular analysis, a single leg was sampled from each collected specimen. Standard *COI* barcodes (658-bp 5’ segment of mitochondrial cytochrome oxidase subunit I) were obtained using primers and protocols described by Shapoval et al. (2017).

**Figure 2.**
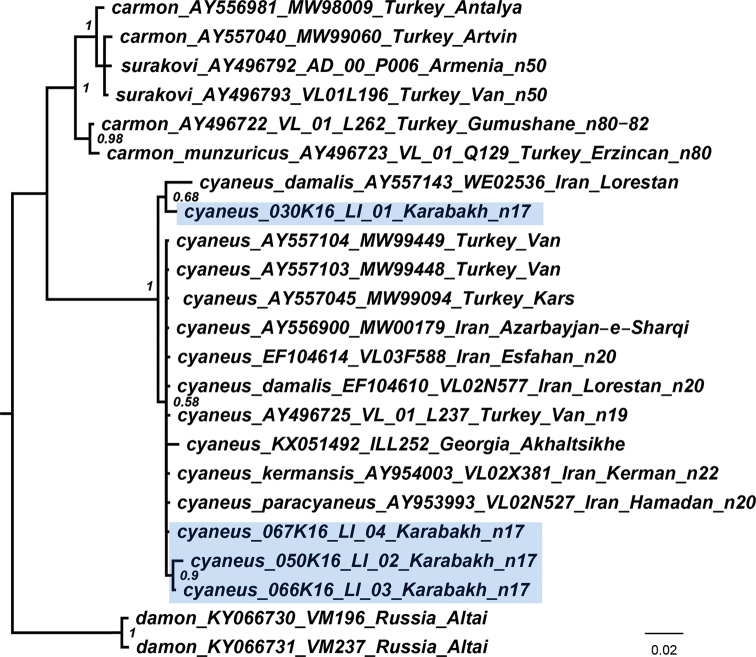
The Bayesian majority rule consensus tree of the analyzed samples of Polyommatus (Agrodiaetus) inferred from *COI* sequences. *Polyommatus damon* (Denis et Schiffermüller, 1775) is used to root the tree. Species and subspecies names, GenBank accession numbers, museum ID numbers, localities and haploid chromosome numbers (if known) are shown to the right of the branches. Bayesian posterior probabilities higher than 0.5 are shown next to the recovered branches.

The Bayesian majority rule consensus tree of the analyzed samples (Fig. 2) was constructed as previously described (Przybyłowicz et al. 2014; Lukhtanov and Iashenkova 2019) using the sequences obtained in this study as well as the published sequences downloaded from GenBank (Wiemers 2003; Kandul et al. 2004, 2007; Lukhtanov et al. 2005; Vishnevskaya et al. 2016). Briefly, sequences were aligned using the BioEdit (Hall 1999) and edited manually. The Bayesian analysis was performed using the program MrBayes 3.2 (Ronquist et al. 2012) with default settings as suggested by Mesquite (Maddison and Maddison 2015): burn-in = 0.25, nst = 6 (GTR + I + G). Two runs of 10,000,000 generations with four chains (one cold and three heated) were performed. We checked runs for convergence and proper sampling of parameters [effective sample size (ESS) > 200] using the program tracer v1.7.1 (Rambaut et al. 2018). The first 25% of each run was discarded as burn-in. The consensus of the obtained trees was visualised using FigTree 1.3.1 (http://tree.bio.ed.ac.uk/software/figtree/).

For chromosomal analysis, the testes were stained with 2% orcein acetic acid for 8–30 days as previously described (Lukhtanov 2019). The stained material was placed in a drop of 40% lactic acid on a glass slide. The testes were macerated with thin pins. The slide was covered with a coverslip and the macerated testes were squashed between the two glasses. Excess lactic acid was removed with filter paper.

Karyotypes were studied in 5 males. Haploid chromosome number (n) was counted at metaphase I (MI), metaphase II (MII) and prometaphase I cells. For determination of karyotype parameters, 79 metaphase plates (MI and MII) of the highest quality and 11 cells at the stage of prometaphase I were selected. Cells in which the chromosomes were not located on the same plane, as well as cells with overlapping or touching chromosomes and/or bivalents, were rejected and not used for analysis. In some cases, diploid chromosome number (2n) was counted in atypical male meiosis which represent a kind of asynaptic meiosis (Lorković 1990; Lukhtanov and Dantchenko 2017; Lukhtanov et al. 2020b).

A Leica DM2500 light microscope equipped with HC PL APO 100x/1,44 Oil CORR CS lens and S1/1.4 oil condenser head was used for bright-field microscopy analysis. A Leica lens HC PL APO 100x/1,40 OIL PH3 was used for phase-contrast microscopy analysis.

## Results and discussion

DNA-barcode analysis demonstrated that the studied samples collected exactly in the type locality and nearby the type locality are almost identical with the previously studied samples collected in Iran and Turkey (p-distance from 0 to 1.6%) (Fig. 2). Polyommatus (Agrodiaetus) cyaneus and P. (A.) carmon species complexes were found to be strongly diverged (p-distance = 6.3%) confirming previous data (Wiemers 2003; Kandul et al. 2004, 2007).

**Figure 3.**
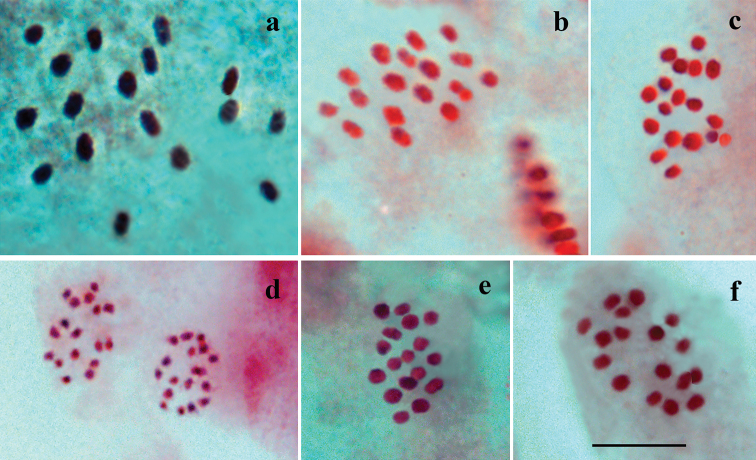
Karyotypes of Polyommatus (Agrodiaetus) cyaneus from Nagorno-Karabakh, Caucasus **a** sample 047K18, Khankendi, prometaphase I, n = 17, phase-contrast **b** sample 047K18, Khankendi, MI, n = 17 **c** sample 047K18, Khankendi, MI, n = 17 **d** sample 030K16, Kanachtala, two MII cells displaying n = 17 **e** sample 050K16, Kanachtala, MI, n = 17 **f** sample 066K16, Kanachtala, MI, n = 17. Scale bar: 10 μ.

In karyotype, at the MI stage, 17 chromosome bivalents were observed in four studied males (Fig. 3a–c, e–f). At the MII stage, 17 chromosome elements were observed (Fig. 3d). The bivalents at the MI and the elements at the MII were found to form a gradient size row in which the largest element was approximately one and a half times larger than the smallest element. In the fifth male, the diploid chromosome number was established as 2n = 34 in male asynaptic meiosis (Table 1). No variation in chromosome number was found.

**Table 1.** Chromosome number in studied samples of P. (A.) cyaneus from its type locality (Nagorno-Karabakh).

Field ID	Lab Id	GenBank#	Chromosome number	Locality
030K16A	L1-01	MW094230	n = 17	near Kanachtala
050K16A	L1-02	MW094231	n = 17	near Kanachtala
066K16A	L1-03	MW094232	n = 17	near Kanachtala
067K16A	L1-04	MW094233	2n = 34	near Kanachtala
047K18A	n/a-	n/a-	n = 17	vicinity of Stepanakert

In terms of chromosome numbers and karyotype structure, the studied populations from Nagorno-Karabakh fit well into the previously described variability within *P. cyaneus* (from n = 16–17 to 22) (de Lesse 1963; Lukhtanov 1989; Lukhtanov et al. 1998). De Lesse (1963), based mainly on his chromosomal studies, divided *Agrodiaetus carmon* (Herrich-Schäffer, 1851) (sensu Forster 1956) into two different species: *A. carmon* with n = 80–82 and *A. cyaneus* with chromosome numbers varying from n = 16 to n=22 in different populations in Iran and Turkey.

Over the next years, the following important additions were made to the taxonomy and cytogenetics of these two species complexes. (i) Chromosome numbers supporting the findings of de Lesse (1963), were established for additional populations (Lukhtanov 1989; Lukhtanov et al. 1998; Kandul et al. 2007). (ii) Polyommatus (Agrodiaetus) carmon was divided in two allopatric, chromosomally diverged species: P. (A.) carmon sensu stricto (n = 80–82) and P. (A.) surakovi Dantchenko et Lukhtanov, 1994 (n = 50) (Lukhtanov and Dantchenko 2002b). (iii) P. (A.) carmon and P. (A.) cyaneus were found as distantly related species complexes, not sister species (Wiemers 2003; Vershinina and Lukhtanov 2017). However, all of the above conclusions were imperfect in terms of zoological nomenclature, since the karyotype of *P. cyaneus* from its type locality was not studied. Our data on topotypes, both in terms of karyotypes and mitochondrial DNA, solve this problem, confirming the taxonomic hypothesis of de Lesse (1963) that P. (A.) carmon and P. (A.) cyaneus as two distinct species complexes.

At the same time, one should note the high chromosomal variability within the taxon, which is now called *P. cyaneus*, as well as the confinement of certain karyotypes to geographic regions. For example, there is a clear tendency that lower chromosome numbers are found in the northern half of the complex’s geographic distribution, and higher ones in the southern half. It is therefore expectable that subsequent studies will shed light on finer taxonomic and phylogeographic structure of this complex.
